# Markers of metabolic endotoxemia as related to metabolic syndrome in an elderly male population at high cardiovascular risk: a cross-sectional study

**DOI:** 10.1186/s13098-018-0360-3

**Published:** 2018-07-21

**Authors:** Ayodeji Awoyemi, Marius Trøseid, Harald Arnesen, Svein Solheim, Ingebjørg Seljeflot

**Affiliations:** 1 Center for Clinical Heart Research, Department of Cardiology, Oslo University Hospital Ullevål, P.O. Box 4956, Nydalen, 0424 Oslo, Norway; 2 Center for Heart Failure Research, Oslo University Hospital, P.O. Box 4956, Nydalen, 0424 Oslo, Norway; 3 Research Institute of Internal Medicine, Oslo University Hospital Rikshospitalet, P.O. Box 4950, Nydalen, 0424 Oslo, Norway; 4 Section of Clinical Immunology and Infectious diseases, Oslo University Hospital Rikshospitalet, P.O. Box 4950, Nydalen, 0424 Oslo, Norway; 5 Institute of Clinical Medicine, Faculty of Medicine, University of Oslo, P.O. Box 1171, Blindern, 0318 Oslo, Norway

**Keywords:** Metabolic syndrome, Gut microbiota, Lipopolysaccharide binding protein, CD14, Innate immunity, Chronic inflammation, Central obesity

## Abstract

**Background:**

Metabolic syndrome (MetS) is a cluster of conditions that conjoined represents a 1.5–2.5 fold increased risk of developing cardiovascular disease (CVD). Recent studies have reported that gut dysbiosis and leakage of bacterial components, may contribute to the metabolic disturbances and systemic inflammation observed in subjects with MetS. Chronic exposure to lipopolysaccharide (LPS) has been shown to induce features of MetS in experimental studies. LPS interacts with the innate immune system, facilitated through LPS-binding protein (LBP) and the co-receptor CD14, both regarded as markers of gut leakage.

**Purpose:**

We investigated whether circulating levels of LBP and sCD14 are associated with the presence of MetS and its components, and further any association with systemic inflammation.

**Methods:**

We examined 482 men, aged between 65 and 75 years, all at high CVD risk. MetS criteria’s according to the US National Cholesterol Education Program Adult Treatment Panel III were met in 182 subjects (38%).

**Results:**

Levels of LBP and sCD14 did not differ between individuals with and without MetS. However, a trend towards increased risk of MetS through quartiles of LBP was observed (p = 0.05). Individuals in the highest quartile (Q4), had an increased risk of MetS (OR = 1.76, 95% CI (1.04–3.00), compared to the lowest quartile (Q1) (p = 0.04). With regard to the separate constituents of MetS, patients who met the waist circumference criterion had significant higher concentration of LBP compared to those who did not (p = 0.04). We also found a weak, but significant correlation between LBP and waist circumference (r = 0.10, p = 0.03). Moderate, yet significant correlations were observed between both LBP and sCD14 and several markers of systemic inflammation (r = 0.1–0.23; p < 0.001–0.04).

**Conclusion:**

The trend for increased prevalence of MetS observed with increasing quartiles of LBP seems to be mainly driven by central obesity in our male cohort. The associations between LBP, sCD14 and systemic inflammation, indicate a potential role of the innate immune system in MetS.

*Trial registration* CLINICALTRIALS.GOV, NCT00764010. Registered 01 October 2008—retrospectively registered, https://clinicaltrials.gov/ct2/show/NCT00764010?term=NCT00764010&rank=1

## Background

The prevalence of metabolic syndrome (MetS) is rapidly increasing in the western world. Its health consequences includes a twofold increased risk of developing cardiovascular disease (CVD), a sevenfold increased risk of developing diabetes mellitus type 2 (T2DM) and a 1.5-fold increased risk of all-cause mortality [1, 2].

There is no uniform definition of MetS, which essentially is a testimony of its complexity. MetS is a cluster of clinical and biochemical conditions which are often found to coexist, thus indicating a common pathophysiological background. The common denominators are central obesity, insulin resistance/glucose intolerance, dyslipidemia and hypertension [3], also known as Norman Kaplan’s “deadly quartet” [4].

The underlying pathophysiology remains unclear and is widely debated. There is, however, compelling evidence that MetS is associated with chronic low-grade inflammation, typically demonstrated by increased levels of C-reactive protein (CRP) and pro-inflammatory cytokines like interleukin-6 (IL-6) and tumor necrosis factor alpha (TNFα) compared to subjects without MetS [5, 6]. Abdominal adipose tissue seems to be an important source of this inflammatory response, and the size and composition of this compartment have been shown to correlate well with the amount of circulating pro-inflammatory cytokines [7].

Translocation of parts of the gut microbiome, and in particular endotoxins or lipopolysaccharides (LPS) to the systemic circulation, has been proposed to be an early trigger of inflammation and subsequent cardiovascular risk [8]. An increase in plasma LPS can occur in healthy individuals after a high fat meal, partly due to co-transportation over the gut wall together with dietary fat by incorporation in triglyceride-rich chylomicrons [9, 10]. Leakage through dysfunctional tight-junctions have also been suggested [11]. Obese individuals tend to have higher levels of circulating LPS, both in fasting conditions and in the postprandial phase [10], and circulating levels of LPS have been reported to correlate with abdominal obesity and glycemic control [12]. In experimental human studies, chronic LPS exposure has been shown to promote systemic insulin resistance and adipose tissue related inflammation [13].

To communicate with the innate immune system, LPS binds to the LPS-binding protein (LBP), which is pivotal for the binding of CD14 and transfer to the Toll like receptor (TLR) 4 complex [14]. Further activation of NF-κB and interferon regulatory factors induces transcription of pro-inflammatory mediators. Blockage of LBP or CD14-binding seems to attenuate the inflammatory effect of LPS in animal studies [15, 16]. Circulating levels of LBP have also been reported to correlate with abdominal obesity and glycemic control [12, 17].

We hypothesize that microbial translocation may contribute to the inflammatory state associated with MetS. We therefore explored any association between circulating levels of LBP and soluble CD14 (sCD14) and the presence of MetS, its components and hyperglycemia. We also explored any association to systemic inflammation.

## Methods

### Study population

The study participants were enrolled in the Diet and Omega-3 Intervention Trial on Atherosclerosis (DOIT) initiated in 1997. The study was designed as a prospective randomized trial [18]. The subjects were all men, aged between 65 and 75 years, deemed at high cardiovascular risk. They were essentially survivors from the Oslo study cohort, conducted in 1972–1977 [19]. The present investigation is a cross-sectional study on baseline data obtained at inclusion. A total of 563 subjects were included in the study. Blood samples from 482 subjects were available for the present investigation.

MetS was classified using the Adult Treatment Panel III (NCEP) definition [20]. The classification requires three or more of the following risk factors: Abdominal obesity defined by a waist circumference > 102 cm in men, triglyceride levels > 1.7 mmol/L, HDL cholesterol < 1.04 mmol/L in men, hypertension defined by blood pressure ≥ 130/≥ 85 and fasting glucose ≥ 5.6 mmol/L. The presence of previously diagnosed hypertension outside the definition of MetS, was defined as systolic blood pressure > 140 and/or diastolic blood pressure > 90 mmHg and diabetes as manifest diabetes and/or fasting glucose > 7 mmol/L. We divided fasting glucose according to the American Diabetes Association definition of normal levels, impaired fasting glucose and diabetes mellitus (≤ 5.5, 5.6–6.9 and ≥ 7.0 mmol/L, respectively). Smokers were defined as current smokers.

### Laboratory methods

Blood samples were obtained at inclusion in fasting condition (> 10 h) by standard venipuncture before daily intake of medication between 08:00 and 10:00 a.m. EDTA blood was separated by centrifugation within 1 h at 2500×*g* for 10 min and plasma was kept stored at − 80 °C until analyses. Serum lipids were determined by conventional enzymatic methods. LPB and sCD14 were analyzed by commercial ELISAs (Hycult Biotech, Uden, the Netherlands and R & D Systems Europe, Abingdon, Oxon, UK, respectively). The inter-assay coefficients of variation were for LBP 8.2% and for sCD14 8.9%. Methods for CRP, IL-6, IL-18 and TNFα have previously been described [21].

### Statistics

All statistics were performed using IBM SPSS statistics version 24.0. Demographic data are given as numbers with proportions or medians with 25, 75 percentiles. As most data were not normally distributed, non-parametric statistics were used. For continuous variables, bivariate Spearman’s correlations were used. To identify differences between MetS vs no MetS and between the different components of MetS, Mann–Whitney U-tests were used. Kruskal–Wallis test was used to examine differences between groups of fasting glucose. Furthermore, we explored the relationship between categorical variables using Pearson Chi square. For trend analysis, we used Mantel–Haenszel test and risk was expressed as Odds Ratio by logistic regression. p < 0.05 was considered statistically significant.

## Results

Baseline characteristics and laboratory data of the total study population are shown in Table 1. As displayed, 38% met the criteria of MetS. Within the criteria of MetS, 88% fulfilled the hypertension criterion, 29% the waist criterion, 40% the hypertriglyceridemia criterion, 10% the low HDL criterion and 56% the impaired fasting glucose criterion. Within the individuals who did not fulfill the definition of MetS (n = 300), 56% had two criteria fulfilled, corresponding to 35% of the total population. Only 6% of these, corresponding to 4% of the total population, had no criteria. Furthermore, there were 16% diabetics, 33% current smokers, 29% with previously diagnosed cardiovascular disease (CVD) and 31% were treated for hypertension (Table 1).

**Table 1:** Baseline characteristics of the total study population (n = 482)

Age	70 (67.5, 72.6)
Metabolic syndrome	182 (38)
Number of criteria fulfilled	
0	19 (4)
1	113 (23)
2	168 (35)
3	111 (23)
4	60 (12)
5	11 (2)
Body mass index (kg/m^2^)	26.5 (24.3, 28.6)
Waist circumference (cm)	98 (92, 103)
Systolic blood pressure (mmHg)	148 (135, 160)
Diastolic blood pressure (mmHg)	84 (91, 77)
Previous hypertension	150 (31)
Previous diabetes mellitus	79 (16)
Previous myocardial infarction	89 (18)
Current smokers	160 (33)
Aspirin	131 (27)
Statins	135 (28)
Antidiabetics	21 (4)
HbA1c (%)	5.6 (5.3, 5.9)
Insulin (pmol/L)	118 (94, 154)
HOMA (units)	4.2 (3.3, 5.7)
CRP (mg/L)	3.27 (1.7, 5.8)
IL-6 (pg/mL)	1.53 (1.00, 2.45)
TNFα (pg/mL)	1.10 (0.78, 1.89)
IL-18 (pg/mL)	274 (212, 350)
LBP (µg/mL)	12.9 (10.4, 15.2)
sCD14 (ng/mL)	1293 (1052, 1515)

Median values (25, 75 percentiles) or numbers (proportions) are given. For abbreviations, see text

### LBP and sCD14 as related to MetS and its components

**Fig. 1:**
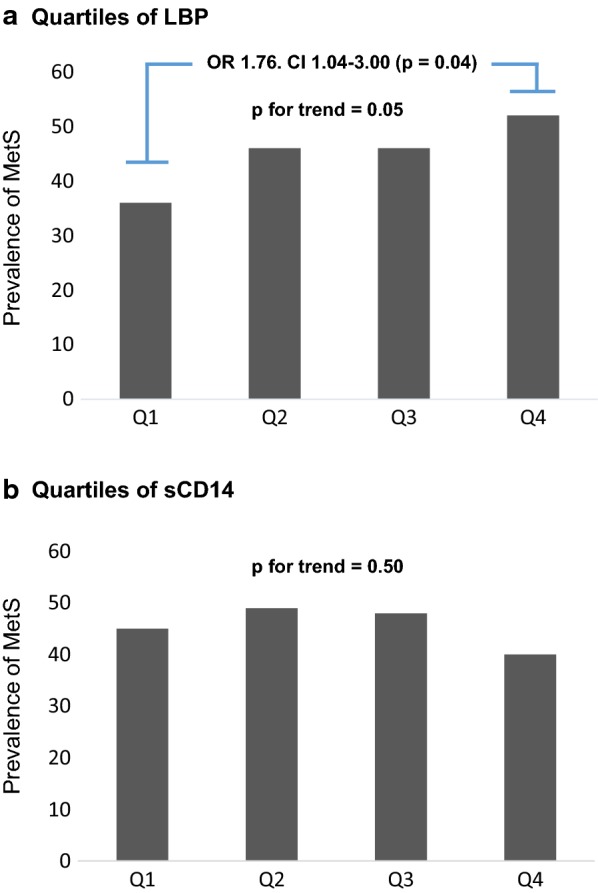
Prevalence of MetS as related to quartiles of LBP (**a**) and sCD14 (**b**) in the total population. P for trend using Mantel Haenszels test. Comparing quartile 4 to 1 of LBP levels, gives an unadjusted Odds ratio of 1.76, p = 0.04 by logistic regression

Neither LBP nor sCD14 levels differed significantly between individuals with MetS compared to those without, although numerically higher levels of LBP in the MetS group was observed (p = 0.11) (Table 2). When dividing LBP and sCD14 into quartiles, we observed a significant trend towards increased prevalence of MetS with ascending quartiles of LBP (p = 0.05) (Fig. 1a). Furthermore, when using the lowest quartile (Q1) as the reference group, subjects in Q4 had an increased risk of having MetS (OR = 1.76, 95% CI (1.04–3.00), p = 0.04). No such trend was observed across quartiles of sCD14 (Fig. 1b).

**Table 2:** Serum concentrations of LBP and sCD14 as related to metabolic syndrome and its separate constituents

	LBP (µg/mL)	p	sCD14 (ng/mL)	p
MetS				
+	13.1 (11.2, 15.5)	0.11	1285 (1057, 1483)	0.71
–	12.7 (10.3, 14.9)		1306 (1053, 1538)	
Waist circumference				
> 102 cm	13.2 (11.2, 15.7)	0.04	1299 (1078, 1494)	0.41
≤ 102 cm	12.6 (10.2, 14.9)		1294 (1033, 1535)	
Blood pressure				
> 130/85 mmHg	12.9 (10.5, 15.2)	0.87	1294 (1051, 1499)	0.36
≤ 130/85 mmHg	13.0 (10.9, 15.0)		1309 (1053, 1627)	
Fasting glucose				
≥ 5.6 mmol/L	12.9 (10.5, 15.3)	0.99	1299 (1065, 1494)	0.95
< 5.6 mmol/L	12.9 (10.6, 15.0)		1293 (1004, 1566)	
HDL-cholesterol				
< 1.04 mmol/L	13.8 (11.1, 15.9)	0.15	1309 (1039, 1594)	0.72
≥ 1.04 mmol/L	12.8 (10.5, 15.1)		1295 (1052, 1518)	
Triglycerides				
> 1.7 mmol/L	13.0 (10.8, 15.4)	0.69	1284 (1061, 1495)	0.87
≤ 1.7 mmol/L	12.7 (10.4, 15.0)		1297 (1030, 1530)	
Fasting glucose				
≤ 5.5	12.9 (10.6, 15.0)	0.99*	1289 (1008, 1558)	0.71*
5.6–6.9	12.8 (10.6, 15.1)		1297 (1072, 1467)	
≥ 7.0	13.1 (10.5, 15.3)		1317 (1056, 1589)	

Median values are given (25, 75 percentiles). P-values refers to differences between groups. * Kruskall–Wallis test. Italic text indicates p-values below < 0.05

Analyzing the separate constituents of the syndrome, concentration of LBP was found to be significantly higher in patients with waist circumference above 102 cm (p = 0.04). This was also observed in the ≤ 5.5 mmol/L glucose subgroup (p = 0.002), but not in other subgroups of plasma glucose. No other differences in LBP levels were observed among the other features of the syndrome (Table 2). sCD14 levels did not differ between the different constituents of MetS.

No trend towards higher levels of LBP nor sCD14 as related to the number of MetS criteria fulfilled was observed in the total population (Data not shown).

When looking at the separate constituents as continuous variables in the whole population, we found a weak, but significant correlation between LBP and waist circumference (r = 0.11, p = 0.02) (Table 3). Furthermore, sCD14 correlated with diastolic blood pressure (r = 0.12, p = 0.01), but not with systolic blood pressure. No significant correlations were observed with regard to levels of fasting glucose, triglycerides or HDL, also when excluding statin users (data not shown).

**Table 3:** Correlations between LBP and sCD14 and metabolic and inflammatory variables in the total population and in subjects with MetS

	In the total population (n = 482)				In subjects with MetS (n = 182)			
	LBP		sCD14		LBP		sCD14	
	r	p	r	p	r	p	r	p
BMI	0.05	0.33	− 0.04	0.34	− 0.06	0.04	0.03	0.70
Waist circumference	0.11	*0.02*	− 0.01	0.89	0.01	0.95	0.13	0.10
Triglycerides	− 0.03	0.56	0.01	0.84	− 0.01	0.86	0.15	*0.04*
HDL cholesterol	− 0.04	0.37	0.02	0.62	− 0.08	0.31	− 0.11	0.15
Systolic blood pressure	0.01	0.78	0.01	0.87	− 0.05	0.51	0.02	0.80
Diastolic blood pressure	0.04	0.36	0.12	*0.01*	− 0.02	0.79	0.07	0.37
Fasting glucose	0.001	0.96	0.01	0.80	− 0.01	0.93	0.10	0.18
Insulin	0.10	*0.04*	0.02	0.74	0.13	0.08	0.02	0.75
HOMA	0.08	0.11	0.03	0.59	0.10	0.18	0.07	0.39
TG/HDL ratio	− 0.01	0.82	0.01	0.86	0.02	0.80	0.15	*0.04*
CRP	0.22	*< 0.001*	0.14	*0.003*	0.17	*0.03*	0.09	*0.23*
IL-6	0.23	*< 0.001*	0.16	*< 0.001*	0.2	*0.001*	0.17	*0.02*
IL-18	0.15	*0.001*	0.18	*< 0.001*	0.17	*0.02*	0.26	*< 0.001*
TNFα	0.15	*0.001*	0.10	*0.04*	0.24	*0.001*	0.06	*0.39*

For abbreviations, see text. Italic text indicates p-values below < 0.05. Subjects with MetS, refers to subjects with 3 out of 5 or more MetS criteria according to the NCEP ATP III definition

Insulin resistance measured by Homeostasis Model Assessment (HOMA) and Triglyceride/HDL ratio (TG/HDL ratio), did not show any correlations with LBP or sCD14. A weak correlation between LBP and serum insulin was observed (r = 0.10, p = 0.04) (Table 3).

Among subjects with MetS, both TG/HDL ratio and triglyceride levels, correlated weakly to sCD14 (r = 0.15, p = 0.04 for both) (Table 3).

### LBP and sCD14 as related to systemic inflammation

Moderate, yet significant correlations were observed in patients with MetS as well as the total population between both LBP and sCD14 and several markers of systemic inflammation, represented by CRP, IL-6, IL-18 and TNF-α as shown in Table 3.

In patients with MetS, CRP, IL-6, IL-18 and TNF-α levels were significantly higher compared to those without in the total population, as previously reported [21].

## Discussion and conclusion

In the present study, concentrations of LBP and sCD14 did not differ significantly in the subjects with MetS vs no MetS although a numerically higher level of LBP was found in the MetS group. We observed, however, a trend towards increasing risk of MetS through quartiles of LBP, in line with previous reports that have shown significant associations between MetS and LBP [22, 23]. The prevalence of comorbidities and the frequent use of medications in both the MetS and the no MetS group, could explain the limited differences in our study, hence the no MetS group cannot be looked upon as a true control group as they may share some of the same metabolic disturbances as seen in MetS. This is further emphasized by the fact that only 6% of the individuals without MetS, did not fulfill any of the MetS criteria and more than 50% had two criteria fulfilled. There are also limited knowledge about how medications affect leakage of LPS from the gut and the expression of sCD14 and LBP. Previous studies have excluded patients on antidiabetics and statins, or use of such medications have not been properly reported [23, 24].

Looking at anthropometric measures in our study population, none of the markers correlated with BMI, however, a significant association was observed between LBP and waist circumference. Central obesity is thought to play a vital role and even accelerate the metabolic and hormonal disturbances observed in this syndrome. The amount of intraabdomial fat has been shown to correlate strongly with serum concentration of LPS as well as bacterial DNA found in intraabdominal fat samples [12]. Thus, although correlations were weak, our finding supports an association and potential link between central obesity and metabolic endotoxemia.

Dyslipidemia in MetS is typically characterized with low levels of HDL and high triglyceride levels. LPS has been shown to be inversely correlated with HDL, thus one could expect an equal relationship between LBP and HDL [25]. In our study, LBP was however, not significantly correlated with HDL or with any of the other lipoproteins in the population as a whole, also when excluding patients on statins, which is known to influence inflammation [26]. In the group with MetS alone, triglycerides were significantly correlated to sCD14.

Glucometabolic parameters are also central in MetS. Hyperglycemia has independently been associated with increased leakage of gut microbial content [27]. We did however, not find any significant differences in LBP nor sCD14 levels between the different ranges of glucose.

Of the glucometabolic parameters, only serum insulin showed a weak positive correlation with LBP in the whole population, while TG/HDL ratio correlated weakly, but significantly to sCD14 in the MetS group. We did not observe any correlations between our markers of gut leakage and HOMA, which is probably due to lack of correlation to fasting glucose. Experimental studies with chronic LPS exposure, typically show decreased insulin sensitivity [13, 28], and several studies have shown correlation between insulin resistance and LBP in humans [17, 29]. Antidiabetics such as sulfonylureas and metformin, whose central mechanisms of action are to stimulate pancreatic beta cells production of insulin and increase glucose sensitivity, respectively, could mask a potential association. However, in our study, the frequency of such drugs was low.

We could also show that the proposed markers of gut leakage, LBP and sCD14, correlate well with downstream mediators of systemic inflammation in the total cohort. For the group with MetS, the correlations were somewhat weaker, which may be due to the reduction in sample size. We have previously shown that IL-6, IL-18, CRP and TNFα, all were significantly higher in the group with MetS compared to those without [21].

LBP is essentially an acute phase protein secreted primarily by hepatocytes and the transcription of LBP is mainly stimulated by IL-1, IL-6 and LPS [30, 31]. Free or membrane-bound LPS is the main ligand of LBP, although it can also bind to other lipopeptides such as Lipoteichoic acid [32], thus not being LPS exclusive. sCD14 can also interact with other pathogen associated molecular patterns (PAMPs) and TLRs, thus not being specific for the LPS–LBP complex [33].

Our population is rather homogenous in terms of being only males and with a narrow age span between 65 and 75 years. Consequently, we were not able to examine differences between sexes and different age groups. Concentrations of LBP are known to increase with increasing age [23].

We have used LBP and sCD14 as surrogate markers of gut related endotoxemia. As stated, both LBP and sCD14 do not exclusively bind to LPS. Conversely, other PAMPs are able to activate the immune system independent of LBP and sCD14. As a result, we are only able to highlight parts of the gut-related activation of the innate immune system.

Furthermore, we did not register daily alcohol intake. Excessive alcohol intake is associated with increased levels of LPS and LBP in serum [34]. Endotoxemia has also been suggested as an important mechanism behind alcohol induced fatty liver. Thus, this may be an important confounder that we are not able to adjust for.

In our study, multiple analyses were conducted using two dependent variables, thereby adding to the risk of Type I errors. However, we decided not to perform Bonferroni correction, because we look upon this study as a hypothesize-generating study, and believe that a correction would be too strict in this case.

To conclude, our study cohort of elderly men cannot confirm that levels of LBP or sCD14 contribute significantly to the low grade inflammation in subjects with MetS. Nevertheless, there was a trend for increased prevalence of MetS with increasing quartiles of LBP which seems to be mainly driven by central obesity. Furthermore, we reaffirm that both LBP and sCD14 are associated with systemic inflammation, indicating a role of the innate immune system in MetS.

## Abbreviations

MetS: metabolic syndrome
CVD: cardiovascular disease
LPS: lipopolysaccharide
LBP: lipopolysaccharide binding protein
T2DM: diabetes mellitus type 2
IL-6: interleukin 6
TNFα: tumor necrosis factor alpha
CRP: C-reactive protein
TLR: toll like receptor
sCD14: soluble cluster of differentiation 14
DOIT: Diet and Omega-3 Intervention Trial on Atherosclerosis
HOMA: Homeostasis Model Assessment
PAMPs: pathogen associated molecular patterns
TG/HDL ratio: Triglycerides/high density lipoprotein ratio

## References

[1] Mottillo S, Filion KB, Genest J, Joseph L, Pilote L, Poirier P, Rinfret S, Schiffrin EL, Eisenberg MJ (2010). The metabolic syndrome and cardiovascular risk a systematic review and meta-analysis. J Am Coll Cardiol.

[2] Wilson PW, D’Agostino RB, Parise H, Sullivan L, Meigs JB (2005). Metabolic syndrome as a precursor of cardiovascular disease and type 2 diabetes mellitus. Circulation.

[3] Kassi E, Pervanidou P, Kaltsas G, Chrousos G (2011). Metabolic syndrome: definitions and controversies. BMC Med.

[4] Kaplan NM (1989). The deadly quartet: upper-body obesity, glucose intolerance, hypertriglyceridemia, and hypertension. Arch Intern Med.

[5] Weiss TW, Arnesen H, Seljeflot I (2013). Components of the interleukin-6 transsignalling system are associated with the metabolic syndrome, endothelial dysfunction and arterial stiffness. Metabolism.

[6] Indulekha K, Surendar J, Mohan V (2011). High sensitivity C-reactive protein, tumor necrosis factor-α, interleukin-6, and vascular cell adhesion molecule-1 levels in Asian Indians with metabolic syndrome and insulin resistance (CURES-105). J Diabetes Sci Technol.

[7] Rexrode KM, Pradhan A, Manson JE, Buring JE, Ridker PM (2003). Relationship of total and abdominal adiposity with CRP and IL-6 in women. Ann Epidemiol.

[8] Lepper PM, Schumann C, Triantafilou K, Rasche FM, Schuster T, Frank H, Schneider EM, Triantafilou M, von Eynatten M (2007). Association of lipopolysaccharide-binding protein and coronary artery disease in men. J Am Coll Cardiol.

[9] Ghoshal S, Witta J, Zhong J, de Villiers W, Eckhardt E (2009). Chylomicrons promote intestinal absorption of lipopolysaccharides. J Lipid Res.

[10] Vors C, Pineau G, Drai J, Meugnier E, Pesenti S, Laville M, Laugerette F, Malpuech-Brugere C, Vidal H, Michalski MC (2015). Postprandial endotoxemia linked with chylomicrons and lipopolysaccharides handling in obese versus lean men: a lipid dose-effect trial. J Clin Endocrinol Metab.

[11] Munkholm P, Langholz E, Hollander D, Thornberg K, Orholm M, Katz KD, Binder V (1994). Intestinal permeability in patients with Crohn’s disease and ulcerative colitis and their first degree relatives. Gut.

[12] Trøseid M, Nestvold TK, Rudi K, Thoresen H, Nielsen EW, Lappegård KT (2013). Plasma lipopolysaccharide is closely associated with glycemic control and abdominal obesity: evidence from bariatric surgery. Diabetes Care.

[13] Mehta NN, McGillicuddy FC, Anderson PD, Hinkle CC, Shah R, Pruscino L, Tabita-Martinez J, Sellers KF, Rickels MR, Reilly MP (2010). Experimental endotoxemia induces adipose inflammation and insulin resistance in humans. Diabetes.

[14] Hailman E, Lichenstein HS, Wurfel MM, Miller DS, Johnson DA, Kelley M, Busse LA, Zukowski MM, Wright SD (1994). Lipopolysaccharide (LPS)-binding protein accelerates the binding of LPS to CD14. J Exp Med.

[15] Le Roy D, Di Padova F, Tees R, Lengacher S, Landmann R, Glauser MP, Calandra T, Heumann D (1999). Monoclonal antibodies to murine lipopolysaccharide (LPS)-binding protein (LBP) protect mice from lethal endotoxemia by blocking either the binding of LPS to LBP or the presentation of LPS/LBP complexes to CD14. J Immunol.

[16] Kim D, Kim JY (2014). Anti-CD14 antibody reduces LPS responsiveness via TLR4 internalization in human monocytes. Mol Immunol.

[17] Moreno-Navarrete JM, Ortega F, Serino M, Luche E, Waget A, Pardo G, Salvador J, Ricart W, Fruhbeck G, Burcelin R, Fernandez-Real JM (2012). Circulating lipopolysaccharide-binding protein (LBP) as a marker of obesity-related insulin resistance. Int J Obes (Lond).

[18] Hjerkinn EM, Seljeflot I, Ellingsen I, Berstad P, Hjermann I, Sandvik L, Arnesen H (2005). Influence of long-term intervention with dietary counseling, long-chain n − 3 fatty acid supplements, or both on circulating markers of endothelial activation in men with long-standing hyperlipidemia 1–3. Am J Clin Nutr.

[19] Hjermann I (1983). A randomized primary preventive trial in coronary heart disease: the Oslo study. Prev Med.

[20] Grundy SM, Cleeman JI, Daniels SR, Donato KA, Eckel RH, Franklin BA, Gordon DJ, Krauss RM, Savage PJ, Smith SC (2005). Diagnosis and management of the metabolic syndrome: an American Heart Association/National Heart, Lung, and Blood Institute Scientific Statement. Circulation.

[21] Troseid M, Seljeflot I, Hjerkinn EM, Arnesen H (2009). Interleukin-18 is a strong predictor of cardiovascular events in elderly men with the metabolic syndrome: synergistic effect of inflammation and hyperglycemia. Diabetes Care.

[22] Jialal I, Devaraj S, Bettaieb A, Haj F, Adams-Huet B (2015). Increased adipose tissue secretion of Fetuin-A, lipopolysaccharide-binding protein and high-mobility group box protein 1 in metabolic syndrome. Atherosclerosis.

[23] Gonzalez-Quintela A, Alonso M, Campos J, Vizcaino L, Loidi L, Gude F (2013). Determinants of serum concentrations of lipopolysaccharide-binding protein (LBP) in the adult population: the role of obesity. PLoS ONE.

[24] Kim KE, Cho YS, Baek KS, Li L, Baek KH, Kim JH, Kim HS, Sheen YH (2016). Lipopolysaccharide-binding protein plasma levels as a biomarker of obesity-related insulin resistance in adolescents. Korean J Pediatr.

[25] Al-Attas OS, Al-Daghri NM, Al-Rubeaan K, da Silva NF, Sabico SL, Kumar S, McTernan PG, Harte AL (2009). Changes in endotoxin levels in T2DM subjects on anti-diabetic therapies. Cardiovasc Diabetol.

[26] Rezaie-Majd A (2002). Simvastatin reduces expression of cytokines interleukin-6, interleukin-8, and monocyte chemoattractant protein-1 in circulating monocytes from hypercholesterolemic patients. Arterioscler Thromb Vasc Biol.

[27] Thaiss CA, Levy M, Grosheva I, Zheng D, Soffer E, Blacher E, Braverman S, Tengeler AC, Barak O, Elazar M (2018). Hyperglycemia drives intestinal barrier dysfunction and risk for enteric infection. Science.

[28] Cani PD, Amar J, Iglesias MA, Poggi M, Knauf C, Bastelica D, Neyrinck AM, Fava F, Tuohy KM, Chabo C (2007). Metabolic endotoxemia initiates obesity and insulin resistance. Diabetes.

[29] Zhu Q, Zhou H, Zhang A, Gao R, Yang S, Zhao C, Wang Y, Hu J, Goswami R, Gong L, Li Q (2016). Serum LBP is associated with insulin resistance in women with PCOS. PLoS ONE.

[30] Geller DA, Kispert PH, Su GL (1993). Induction of hepatocyte lipopolysaccharide binding protein in models of sepsis and the acute-phase response. Arch Surg.

[31] Dentener MA, Vreugdenhil ACE, Hoet PHM, Vernooy JHJ, Nieman FHM, Heumann D, Janssen YMW, Buurman WA, Wouters EFM (2000). Production of the acute-phase protein lipopolysaccharide-binding protein by respiratory type II epithelial cells. Am J Respir Cell Mol Biol.

[32] Schroder NW, Morath S, Alexander C, Hamann L, Hartung T, Zahringer U, Gobel UB, Weber JR, Schumann RR (2003). Lipoteichoic acid (LTA) of *Streptococcus pneumoniae* and *Staphylococcus aureus* activates immune cells via toll-like receptor (TLR)-2, lipopolysaccharide-binding protein (LBP), and CD14, whereas TLR-4 and MD-2 are not involved. J Biol Chem.

[33] Akashi-Takamura S, Miyake K (2008). TLR accessory molecules. Curr Opin Immunol.

[34] Uesugi T, Froh M, Arteel GE, Bradford BU, Wheeler MD, Gabele E, Isayama F, Thurman RG (2002). Role of lipopolysaccharide-binding protein in early alcohol-induced liver injury in mice. J Immunol.

